# Capacity-based legislation in Norway has so far scarcely influenced the daily life and responsibilities of patients’ carers: a qualitative study

**DOI:** 10.1186/s12888-023-04611-4

**Published:** 2023-02-20

**Authors:** Nina Camilla Wergeland, Åshild Fause, Astrid Karine Weber, Anett Beatrix Osnes Fause, Henriette Riley

**Affiliations:** 1 Division of Mental Health and Substance Abuse, University Hospital of North Norway, Tromsø, Norway; 2 Department of Health and Care Sciences, Faculty of Health Sciences, UiT The Arctic University of Norway, Tromsø, Norway; 3 National Competence Center for Community Mental Health (NAPHA), Trondheim, Norway; 4 Elden Law Firm, Tromsø, Norway

**Keywords:** Carer, Family-carer, Capacity-based legislation, Coercion, Community treatment order, Patient autonomy, The Norwegian mental health act

## Abstract

**Background:**

When capacity-based mental health legislation was introduced in Norway in 2017, there was concern about the consequences of change in the law for patients’carer whose community treatment order was revoked as a result of being assessed as having capacity to consent. The concern was that the lack of a community treatment order would increase carers’ responsibilities in an already challenging life situation.

The aim of this study is to explore carers’ experiences of how their responsibility and daily life were affected after the patient’s community treatment order was revoked based on capacity to consent.

**Method:**

We conducted individual in-depth interviews from September 2019 to March 2020 with seven carers of patients whose community treatment order was revoked following assessment of capacity to consent, based on the change in the legislation. The transcripts were analysed with inspiration from reflexive thematic analysis.

**Results:**

The participants had little knowledge about the amended legislation, and three out of seven did not know about the change at the time of the interview. Their responsibility and daily life were as before, but they felt that the patient was more content, without relating this to the change in the law. They had found that coercion was necessary in certain situations, which made them worry whether the new legislation would make it more difficult to use coercion.

**Conclusion:**

The participating carers had little or no knowledge of the change in the law. They were involved in the patient’s everyday life as before. The concerns prior to the change about a worse situation for carers had not affected them. On the contrary, they found that their family member was more satisfied with life and the care and treatment provided. This may suggest that the intention of the legislation to reduce coercion and increase autonomy was fulfilled for these patients, without resulting in any significant change in carers’ lives and responsibilities.

## Background

The introduction of capacity-based legislation in mental healthcare in Norway on 1 September 2017, see Table 1, was extensively debated. The new legislation was expected to reduce the number of Community treatment orders (CTOs) [3], because compulsory maintenance treatment for patients with severe mental illness could not now be continued if they had capacity to consent [1, 3]. Previous studies have shown that carers found that CTOs provided stability for both patient and family, ensuring follow-up treatment from healthcare services [8, 9].

**Table 1:** Capacity-based legislation in Norway

Capacity-based legislation in Norway Capacity-based legislation was introduced on 1 September 2017 as an amendment to the Norwegian Mental Health Act [1] in order to strengthen patient autonomy in accordance with the United Nations Universal Declaration of Human Rights and the Convention on the Rights of Persons with Disabilities [2, 3]. Several European countries and Australia have also introduced various forms of capacity-based legislation [3–5]. The change in the law represents a shift from decisions to use coercion based on diagnosis to a focus on the patient’s autonomy, i.e. a capacity-based criterion [3, 5, 6]. A new condition in Section 3–3 of the Mental Health Act §3–3 [1] is: The patient lacks capacity to consent, cf. the Patient Rights Act §4–3 [7], which states that capacity to consent may be partly or wholly invalidated if the patient, due to a physical or mental disorder, dementia or an intellectual disability, is clearly unable to understand what the consent implies. The new condition shall not apply in cases of imminent and serious danger to the patient’s own life or the life or health of others [1]. A decision to use coercion is based on a clinical assessment; the patient’s capacity to consent is assessed by the patient’s therapist, who must be a psychiatrist or specialist clinical psychologist [1]. Decisions on the use of coercion can be reviewed by a control commission, and the commission’s decision may be submitted for judicial review [1].	

When the new legislation was introduced, carer associations and healthcare personnel were concerned about possible consequences for patients and carers [3, 10–13]. Without a CTO, the concern was that patients would refuse treatment and follow-up care, or any healthcare services. During initial work on the legislation and in the media, fears emerged that patients would deteriorate and that carers’ responsibilities would increase in an already challenging situation [3, 10–13]. However, carer associations supported the new legislation because they believed that a reduction in coercion was necessary, but they also pointed out that for the legislation to work as intended, more flexible healthcare for patients and separate support for carers were necessary [13].

Carers of people with co-occurring severe mental illness and substance abuse are not a uniform group. Under Norwegian law, the person designated by the patient as the “closest carer” is entitled to receive specific information about the patient’s condition and health care if the patient consents to this [14]. When the patient is unable to decide on the closest carer, the person with the most stable contact with the patient will receive these rights [14]. If the patient lacks capacity to consent, the closest carer is entitled to be involved with the patient in treatment decisions and to receive the same information about the patient’s condition as the patient, unless otherwise determined [7].

The Mental Health Action Plan 2013–2020 of the World Health Organization calls for increased cooperation with carers [15], and several nations have made efforts to strengthen carers’ position and rights through health policy guidelines [16]. Studies show that family involvement can have positive effects for patients, clinicians and carers [16, 17]. However, studies from several Western countries show that carers are still little involved in collaboration [18, 19] they do not often have the opportunity to share important information and receive scant support from the healthcare system to handle their responsibilities [8, 16, 20–22].

No studies on carers’ experiences of the introduction of capacity-based legislation have been published in Norway. The aim of this study is to explore carers’ experiences following the revocation of a patient’s CTO based on capacity to consent.

## Method

### Design

This study has a qualitative design using in-depth interviews to explore carers’ experiences following the introduction of capacity-based mental health legislation. It is sub-study three in a larger study that examined the experiences of patients [23] and healthcare personnel [24] with the change in the legislation. The research question of the present study is *What are the experiences of patients’ carers following the change in the legislation and how has the change affected their daily life and perceived responsibility as carers?*

The participants in the various sub-studies formed triads, where patients in the first sub-study chose which relatives would be invited to participate in the present study. The study was conducted in the northernmost health region of Norway.

### Carer’s involvement

When preparing the project proposal, three members of the research team (NCW, AW and HR) conducted four focus group interviews to gain insight into different perspectives, expectations and opinions about the change in the law. One of the focus groups consisted of carers of patients who were or had been under a CTO. The participants were asked what they thought was important to explore in the study, with the aim of including their views in the design of the research questions and the interview guide.

At the beginning of the study, a peer group was established; of the six members, three had experience as carers of a patient under a CTO. In the first two meetings, we discussed the implementation of the study; the members made suggestions on recruitment, conducting interviews and the interview guide. Two members of the group participated in the analysis.

### Recruitment

Inclusion criteria for the participants was: Carers of patients with severe mental illness whose CTO was revoked following assessment of capacity to consent when capacity-based legislation was introduced in 2017. Ten of the patients who participated in the first sub-study [23] gave consent for one of their carers to be interviewed in the present study. Nine of these carers were contacted by telephone by the interviewer (NCW), given information about the study and recruitment, and invited to participate. The tenth carer could not be invited and two interviews that had been agreed on could not be conducted due to COVID-19 and the long time of “shutdown” in Norway. All those invited agreed to participate and were sent written information and a consent form by e-mail. None withdrew from the study.

### Participants

Seven participants were interviewed in this study; they were all carers of patients with severe mental illness, and in two cases concurrent substance abuse. All participants had been in a close relationship with the patient for a long time, four as parents and three as partners or in another close relationship. Most lived near the patient, one lived with the patient, while one lived fare away. They were in the age group 40–70 years, two of the seven were men, while two were retired and five were in full-time employment.

### Interviews

Interviews took place in a hospital, a hotel, and the participants’ home or workplace from September 2019 to March 2020, i.e. at least 2 years since the change in the law. They lasted from 60 to 90 minutes and were audio recorded and subsequently transcribed and anonymized. The first author conducted all the interviews and after each interview made notes about her experience of the interview and the context.

The interview guide consisted of three main parts with questions and cues. The introductory questions dealt with the presentation of the participant, relationship to the patient and the illness trajectory. In the main part of the guide, participants were asked about their knowledge and experience of the change in the law and its significance for the patient’s treatment and follow-up care. They were also asked about any changes to their lives and their cooperation with the patient and clinicians after the CTO was revoked. The last part of the interview guide contained rounding off questions and questions about how they felt about being interviewed.

### Analysis

The approach is hermeneutically inspired. This implies that the data were generated in a dialogue between the participants’ narratives and the researchers’ understandings [25, 26]. The analysis of the interview data was also inspired by reflexive thematic analysis as developed by Braun and Clarke [27, 28]. The fact that this is the third part of the larger study was of vital importance to the research team’s reflections on the participants’ narratives during the analysis. The knowledge gained from the first two sub-studies on experiences of patients and healthcare personnel was included in much of the interviews and in the data analysis and interpretation in this study.

The first phase of analysis as described by Braun and Clarke [28] consists of the researcher’s familiarization with the data. The first author conducted all the interviews, participated in parts of the transcription and listened to the audio recordings several times. When reading the interviews, she focused on each one as a whole, aiming to gain insight into the main issues for the participants. She noted ideas, comments and questions to the data in the margins while reading. This was also done in the next reading, where the focus was on individual statements or passages, and how these could be understood in the light of an overall understanding. A number of statements were highlighted. Reflections were written down on how the statements were understood and associations with other participants’ statements, in addition to brief notes on what the first author felt was the essence of the participants’ narratives.

Once the first author was well acquainted with the data, the second phase of coding commenced [28]. The NVivo software was used for the coding. The statements coded were those that could provide insight into the participants’ life situation, daily life and history with the patient, as well as those that more directly answered the research question. The coding was thus not clearly defined by the research question, but included e.g. the onset of the illness in order to enhance understanding of the duration and progression of the role of the carers. Coding took place in several rounds in order to find precise labels for the codes. It was also necessary to adjust the content of the codes when they were too general, too narrow or too specific. Several attempts were made to sort the codes into groups that seemed to contain related elements. Braun and Clarke [28] point out that preliminary ideas for themes should be kept open to make room for new ideas. However, one theme (little or no knowledge of the change in the law) took shape at an early stage through various codes that nuanced the content such as *not knowing about* and *lacking information*. Preliminary ideas for themes and overviews of codes were presented to the other researchers in the team and later to two of the members of peer group to provide a broader basis for reflections and understandings of the data. In the third phase, the preliminary themes and code groups were visualized in an NVivo map to provide an overview and to assist in finding patterns and meaning across the interviews. Some of the preliminary themes did not answer the research question and some were merged.

Phase three merged with phase four, as they both consisted of developing themes. Three of the resulting themes passed the quality test in Braun and Clarke’s fifth phase [28], which showed that the themes were clear, well defined and unique, and contributed to the study’s overall analysis related to the research question. The names of the themes were changed a number of times even after the results section had been written and the writing of the discussion had started in an attempt to find suitable, precise and informative names. The final themes were as follows: 1) little or no knowledge of the change in the law, 2) responsibility, cooperation and daily life are unchanged, and 3) coercion is felt to be necessary.

### Ethics

This study was assessed by the Northern Norway Regional Ethics Committee (REK Nord), REK No. 2018/1659, and approved by the data protection officer of the University Hospital of North Norway.

All participants received oral and written information about the study. They were also informed about voluntary participation and the possibility to withdraw from the study at any time before the data were included in the analysis, without giving any reason.

The participants were given pseudonyms and their gender and characteristics may have been changed for the purpose of anonymization without any effect on the content of the study.

The interview topics may have been difficult to talk about. The participants were asked to talk about their family member’s often long and challenging illness, which meant a difficult situation for the whole family. Several of the participants were fatigued and some may have agreed to participate because they felt obliged to do so. The interview may have been perceived as intrusive and was therefore conducted with consideration and empathy. The interviewer provided a safe space for the participants to talk about their experiences of responsibility. Several started by saying that they maybe should have come better prepared to the interview. They were assured that good preparation was not relevant to the purpose of the interview, and that they should only talk about what they wanted to share. However, several found it difficult to talk about their challenging life situation and memories they had tried to forget. Some cried, but were pleased to be able to talk about their experiences and to contribute to research.

## Results

The results presented below must be seen in the light of the participants’ challenging life situation.

The participants said that having a close family member with a severe mental illness who had been under a CTO had been very demanding and had affected the daily life of the entire family. They constantly worried about the patient and what the future would bring. Heidi put it this way:

*“It’s a really really big role being Simon’s mother. I haven’t had a holiday for many, many years, I’m so afraid of being away from him if something happens to him.”*

Heidi and Berit had mostly had sole responsibility for their sick children. Berit said that she was in a new and less demanding phase as a carer at the time of the interview, but described her responsibility over many years as follows:

*“Well, it hasn’t exactly been a walk in the park, I can tell you. But I’ve kept going. I’ve coped, but it’s been pretty tough at times. To be a mother in this situation.”*

### Little or no knowledge of the change in the law

The participants had limited or no knowledge of the change in the legislation at the time of the interview. Three of them said that healthcare personnel had spoken to them about the change. They were not sure about who had informed them, but thought it was the patient’s therapist or the staff who provided daily care. Several had been told that the patient’s CTO had ended, but did not realize that it was due to a change in the law. Two of the participants had heard about the change on television or radio. Berit explained:

*“I love watching TV and it was on the news about the new law that had come.”*

The two remaining participants were not aware of the change at the time of the interview, and when asked if she had received information about it, Ella replied:

*“No, this is the first time I’ve heard about it (in the interview).”*

Several participants were surprised that the patient’s CTO had been revoked because they had not noticed any improvement in the patient’s condition or functioning. They had not understood that the patient had been assessed as having capacity to consent and could therefore no longer be under a CTO. Before the CTO was revoked, two participants had been asked if they agreed, which they both did. However, one of them felt that her daughter’s CTO should have been revoked at a much earlier stage.

Berit felt that her daughter was happier and trusted her therapists more over the past 2 years. She had thought that these changes were because her daughter had gained more experience with the disorder and had accepted the need for treatment. Berit had heard about the change in the law on the TV news, but had not thought that the new legislation and greater autonomy could be linked to her daughter’s increased satisfaction until during the interview. When we talked about the change, Berit reasoned as follows:

*“I haven’t thought about it. But it might be because of that (the change in the law)… that she trusts the therapists much more now. Perhaps it’s the change in the law, she’s felt like she has more influence, she’s got the right to decide her own treatment and her own life in all this. It could be. I hadn’t thought much about that until you… But it may well be true. Because she’s much happier and well, all in all…”.*

Two other participants also said that their children were more satisfied with the treatment they had received in the past 2 years since the CTO was revoked.

### Responsibility, cooperation and daily life are unchanged

The treatment and care provided by primary healthcare services were much the same as before the CTO was revoked. All patients received medication treatment, two had outpatient treatment and most received daily follow-up care. Two of the participants were surprised that their family member accepted the same treatment and care as under the CTO. Five of the participants’ family members accepted the treatment and care voluntarily, while two were under a new CTO at the time of the interview. The participants said that they found it necessary to take the same responsibility for the patient as before.

Several participants stated that their burden of responsibility varied according to the patient’s condition and their perception of how well the healthcare services were functioning. As an example of their responsibilities, several participants found it necessary to clear out rubbish and used needles from their child’s room or make sure that he or she took a shower more often. The participants’ perception of the commitment, competence and continuity of the healthcare staff determined how much responsibility they needed to take. Heidi, whose son lived in staffed housing, felt that many staff could be better at communicating with residents in order to provide help. She said:

*“He’s supposed to get the help he needs to tidy up his room in housing with 24-hour staffing, but I can see he’s not getting it. He’s been given over 30 hours a week by the social services, they should help him to tidy up and... but there’s quite a big conflict between me and this housing. I’ve told them, ‘You’re not doing your job’, and they say, ‘But he doesn’t want to’… So I say, ‘Well, how do you ask him then?’, and then I say, ‘If you get to know Per properly, you can ask him in a way that makes him say yes’. And it’s also about building relationships... if he gets a good relationship with someone there, it’s often with people who disappear again.”*

However, four participants reported that both they and the patient thought that healthcare services had improved, but this was against a background of many years of different kinds of treatment from the hospital, the mental health centre and primary health care within a CTO framework. Despite this, the participants were unable to link these experiences to the change in the law. Evy said:

*“Now I feel that the system around him is working. So that... I can sort of just be his mother and I don’t have to be a kind of helper as well.”*

One participant felt that the care provided was still inadequate; there was a large turnover of staff in the housing and a reduction in the hours allotted to care after the CTO was revoked.

### Coercion is felt to be necessary

Three of the participants who were sceptical of the change in the law when they heard about it were now worried about whether it would be more difficult to intervene using coercion if necessary. Heidi, whose son was under a CTO at the time of the interview, felt that it was right to terminate the CTO for her son when the law was changed. She wanted her son to have the chance to take on more responsibility when he was able to. However, she had also felt that a CTO was a necessary framework for treatment and follow-up care that would improve the lives of both patient and family. She said:

*“I think in relation to... well, you know, there are all these admissions and there’s much less of that since he was put on a CTO and getting involuntary medication... When he’s on a CTO and he’s medicated, things are more stable for all of us.”*

Several participants found that a CTO had led to a stable situation for themselves and their family member. They pointed out that a CTO prevented the sudden discontinuation of medication, and reduced admissions to hospital and the unpleasant situations that involuntary admission often involved.

All the participants felt that coercion was necessary to ensure adequate help in certain conditions and situations. They had experience of situations where it was necessary to use force to intervene to prevent serious consequences for the patient and the family as a whole, especially in the early stages of the illness. The participants described living in great uncertainty at times, and several had been threatened by the patient while they were waiting for help to arrive. In such situations, the CTO reassured them that their family member had easier access to treatment from a specialist at the hospital.

One participant, Thomas, was sceptical when the CTO was revoked, because he felt that his relative had limited insight into his illness, and was incapable of taking responsibility for his own health and accepting help. When in fact things turned out well, Thomas suggested two reasons for this. Firstly, his relative had a safe and stable environment in which he received the same healthcare as before from competent professionals. Thomas put it like this:

*“I’d say he’s doing fine now…. as soon as his world is unstable, either he gets less medication, or things change... well, then he gets worse and more unstable again. But as long as he knows what’s going to happen every day, he functions very well. As long as he has a secure framework, and he gets to keep Anna (as his primary contact), I think that’s really important for him to feel ok, she knows him very well and handles him incredibly well, it’s good to see.”*

Secondly, Thomas thought that his realtive was easier to help now that he had grown older with a weaker body and reduced health as a result of his illness and long-term use of psychotropic medications.

The participants expressed a need for a safe and stable situation for the patient and themselves, but they were also interested in voluntary care and treatment when the patient’s condition allowed it.

## Discussion

This study explores carers’ experiences of how the introduction of new legislation on lack of capacity to consent regarding the use of coercion affected their lives and responsibilities as carers. The results show that the participants’ responsibilities and daily life had not changed significantly, but they found that the patient’s condition had improved. The participants had little or no knowledge of the change in the law and its significance. They wished to point out that coercion had been necessary in certain situations.

### The significance of the change in the law

The participants had varying knowledge of the CTO scheme, even though their family member had previously been under a CTO for a long time. Their poor knowledge of the change in the law suggests that they did not know much about the Mental Health Act either before or after the change. Stensrud [9] found in his study that carers of patients under a CTO focused on practical everyday life and effects of the treatment, and were less concerned about coercion. The particular legal terminology and logic made the change difficult to understand, and it may well be natural for carers to feel that the legal aspects are the responsibility of the healthcare services. Carers’ lack of awareness of the change in the law may have been due to the prolonged and complex burden of being close relatives of a patient with severe mental illness, making their lives difficult and leaving them exhausted. Several other carers’ stories in the Norwegian media [10, 11, 29] and in research [22, 30] confirm the participants’ narratives about their burden of responsibility. Having a family member with severe mental illness leaves little energy to study legislation.

Healthcare personnel are responsible for providing advice and information to carers in a clear and comprehensible form [14]. It was probably difficult for clinicians to provide clear information to carers, given the uncertainty in the period following the new law as to how it should be interpreted and practised [24]. However, several studies have shown that poor information, training and involvement of carers are not unusual [17–19, 30]. Healthcare services often lack adequate procedures for the involvement of family members, and leave such work to the personal initiative and competence of individual health workers [17, 20, 30]. This is despite the fact that studies show that involving and supporting carers has a positive effect on treatment quality [17], can reduce the risk of relapse [31–34] and can improve carers’ own health [35, 36].

The new legislation had changed little in the lives of the participants. They had continued their engagement and great responsibility in the life of their family member. The participants found that the patient was more stable with well-established healthcare services at the time of the interview, unlike the first years of the illness. Nevertheless, they still worried about their family member’s future. The participants had previously felt a need for more continuity and competence among healthcare providers because of the event of changes in the patient’s condition and need of a reassured living situation. Some of them had experienced reduced healthcare services and that it became more difficult to admit the patient to hospital, without the framework of a CTO. A decrease in help from the healthcare system after a CTO has ended has been confirmed in other studies [8, 9], and this may have made the participants worry that the change in the law would make the threshold for coercion too high if the patient’s condition deteriorated. One study found that healthcare personnel believe that CTOs makes a difference for patients’ rights and facilitate adequate care provision [37].

In contrast to this, studies of the experiences of patients and clinicians with the change in the law and termination of a CTO show that patients were offered and accepted the same healthcare services [23], and that healthcare staff to a greater extent than previously adapted treatment and care to the patient’s preferences with a focus on the patient maintaining or regaining autonomy [24]. The amendment to the Mental Health Act still allows for involuntary treatment when patients lack capacity to consent and to receive necessary healthcare, or when patients are considered to represent a risk to their own life or the life and health of others [1].

The concerns expressed prior to the change in the law about a worse situation for relatives had not affected the participants in this study at the time of the interview. Most of them, on the contrary, had found that their family member seemed to be more satisfied with life and healthcare services. This suggests that the aim of the legislation to reduce coercion and increase autonomy was fulfilled for the patients, but without significantly changing the carers’ daily life and responsibilities.

Our findings support there is a need for more studies on implementation of research on family involvement for patients with severe mental illness to prevent and reduce use of coercion. There is a lot of knowledge which we need to put to use to improve family involvement practices.

### Strengths and weaknesses

This study forms part of a larger study in which we explore experiences from different perspectives in connection with the assessment of patients as capable to consent leading to the revocation of CTOs [23, 24]. This design gave us a variety of perspectives on the same treatment path and on the change in the law. The interviews and analyses in the present study were influenced by the research team’s experience from the first and second parts of the larger study. This experience has expanded our understanding and enabled us to see connections and coherence in a way that would have been impossible without a triad design. At the same time this required us to be aware of the influence the knowledge from sub-studies one and two possible had on our preconception. We have listened to and read the participants statements carefully in this third sub-study, with an aim to let them present themselves and to perceive their stories and their versions of the situation.

The interviews were conducted in 2019 and 2020. At that time, the participants had limited knowledge of the change in the law and the results might have been different if the new legislation had been implemented for several years. Feedback from carers in the peer group suggests that it took time to understand the significance of the content of the amended legislation.

This study had a limited number of participants. A greater number would probably have enhanced the diversity of the study. A further three interviews were planned but had to be cancelled due to the ongoing COVID-19 pandemic. The study was conducted in a limited geographical area where one hospital is responsible for all involuntary mental health care, which may mean that the findings are influenced by local practice.

## Conclusion

The study participants were carers of patients with severe mental illness whose CTO was revoked following assessment of capacity to consent when capacity-based legislation was introduced in 2017. They had little or no knowledge of the change in the law at the time of the interview. We found that only a minority had heard of the change, and that these had little understanding of its significance. A further finding was that the change in the law had no great influence on relatives’ responsibilities. The participants were just as involved in the life of their family member and their daily life was little changed. At the same time, several participants found that the patient was more satisfied and independent, but did not relate this to the legislation. Based on their experience of the patient’s severe mental illness and fluctuating condition, the participants felt that coercive intervention could be necessary in certain situations, and were therefore worried that the change in the law would make this more difficult to implement.

## Data Availability

The datasets generated and analysed during the current study are not publicly available in order to protect the anonymity of the participants, but are available from corresponding author on reasonable request.

## Abbreviation

CTO: Community treatment order
